# Isolation, Identification, and Characterization of *Listeria* from Municipal and Dairy Farm Wastewater

**DOI:** 10.3390/microorganisms14102216

**Published:** 2026-10-02

**Authors:** Caitlyn Quinn, Peter M. Muriana

**Affiliations:** 1 Robert M. Kerr Food and Agricultural Products Center, Oklahoma State University, Stillwater, OK 74078, USA; caitlyn.quinn@okstate.edu; 2 Department of Animal and Food Sciences, Oklahoma State University, Stillwater, OK 74078, USA

**Keywords:** *Listeria monocytogenes*, wastewater, PCR, Pathogenic Island, chromogenic agar

## Abstract

Wastewater systems can serve as reservoirs and dissemination pathways for *Listeria* spp., yet comparative data from municipal and agricultural wastewater remain limited. The objective of this study was to isolate *Listeria monocytogenes* from municipal wastewater and dairy-farm runoff for use as enrichment hosts in a subsequent and separate study on the isolation of bacteriophage from the same wastewater streams. During this process, additional *Listeria* spp. were recovered and characterized along with *L. monocytogenes.* Identification of *Listeria* sp. was accommodated using multilocus sequencing, LIPI-1-targeted PCR, and phenotypic confirmation. Wastewater samples were filtered, enriched with Demi-Fraser broth, treated with anti-*Listeria* magnetic beads, and plated on MOX agar. Bead-captured *Listeria* were later plated on *Listeria* Brilliance Agar to differentiate phospholipase-positive *L. monocytogenes*. Putative isolates were characterized by sequencing of 16S rRNA, *sigB*, and *iap* genes, and by PCR detection of four *Listeria* Pathogenicity Island 1 (LIPI-1) markers (*hly*A, *inl*A, *act*A, *prf*A). Using this combined approach, 27 *Listeria* isolates were recovered from 21 wastewater samples during a 6-week sampling period: 13 samples of municipal wastewater yielded 5 isolates of *L. monocytogenes* (38% prevalence rate) and 11 other *Listeria* sp. (85% overall prevalence rate; 6 *L. innocua*, and 5 *Listeria* sp.); 8 samples of dairy wastewater yielded 2 isolates of *L. monocytogenes* (25% prevalence) and 9 other *Listeria* sp. (100% overall prevalence rate; 8 *L. innocua* and 1 *Listeria* sp.). Seven isolates from municipal and dairy wastewater were confirmed as *L. monocytogenes* based on concordant *sigB* and *iap* gene sequence identities, LIPI-1 gene presence, and phospholipase-positive reactions on chromogenic agar. Six isolates yielded discordant results across the multilocus sequencing markers and could not be confidently assigned to a species and were designated *Listeria* sp. These findings highlight the value of integrating chromogenic media with multilocus genetic markers for accurate identification of *L. monocytogenes* in complex environmental matrices.

## 1. Introduction

*Listeria monocytogenes* is a major foodborne pathogen associated with severe invasive disease, high hospitalization rates, and substantial mortality, making its environmental ecology a critical component of food-safety risk assessment [1]. Non-pathogenic *Listeria* spp., including *L. innocua*, are widespread in soil, water, and agricultural environments and often serve as ecological indicators of conditions that may also support *L. monocytogenes*. Because both pathogenic and non-pathogenic *Listeria* species persist in moist, nutrient-rich environments and can form biofilms on diverse surfaces, wastewater systems represent plausible reservoirs and dissemination pathways for these organisms. This study was initiated with the objective of isolating *Listeria monocytogenes* from municipal and agricultural wastewater as host strains for downstream bacteriophage enrichment. Because the sampling structure and dataset were not designed to support robust prevalence estimates, the present report focuses solely on species identification and recovery of *L. monocytogenes*.

Municipal wastewater integrates microbial inputs from households, food establishments, healthcare facilities, and environmental stormwater runoff, while dairy-farm wastewater contains fecal material, feed residues, bedding, and milking-parlor effluent. These systems aggregate microbial signals from large and heterogeneous upstream populations, making them valuable matrices for detecting environmentally persistent organisms such as *Listeria* [2]. Wastewater-based surveillance has been widely recognized as an efficient, population-level monitoring tool capable of detecting pathogen biomarkers shed into sewer systems [3]. During the COVID-19 pandemic, it demonstrated the ability to detect viral pathogens before increases in clinical cases were observed [4]. These systems have shown that wastewater monitoring can provide early warning signals, reveal spatial and temporal trends, and capture pathogen circulation even when clinical testing is limited.

The same principles that make wastewater surveillance effective for viral pathogens are applicable to environmentally persistent foodborne bacteria. Wastewater-based detection has been successfully used for *Salmonella*, *Campylobacter*, *Escherichia coli* O157:H7, and other enteric pathogens in various surveillance programs [5]. Extending these approaches to *Listeria* spp. is particularly relevant given the organism’s environmental resilience, its ability to survive in cold and nutrient-poor conditions, and its recognized presence in agricultural runoff and surface waters [6,7]. Despite this relevance, systematic surveillance of *Listeria* in municipal and agricultural wastewater remains limited, and few studies have compared species distribution and *L. monocytogenes* occurrence across these distinct wastewater environments.

Understanding the prevalence, diversity, and genetic characteristics of *Listeria* spp. in wastewater can provide insight into environmental dissemination pathways, potential contamination routes into food-production systems, and upstream sources contributing to pathogen circulation. Such information complements existing public-health surveillance systems for *Listeria* infections, which traditionally rely on clinical case reporting and foodborne-disease monitoring. Wastewater-based detection may therefore serve as an early-warning or supplementary surveillance mechanism, particularly in regions with intensive agricultural activity or high-risk food-processing operations [2,8].

To address these gaps, *Listeria* spp. were isolated and characterized, including *L. monocytogenes*, from municipal and dairy-farm wastewater (the dairy farm experienced the death of 6 aborted fetuses from 9 pregnant dairy cows in the preceding 3 months due to *L. monocytogenes*). The approach involved a multilocus genetic identification strategy addressing 16S rRNA sequencing [9,10], the *sigB* stress-response gene [11,12], the *iap* invasion-associated protein gene [13], and four *Listeria* Pathogenicity Island 1 (LIPI-1) markers [14,15]. These loci provide complementary taxonomic and virulence-associated resolution: 16S for broad genus-level identification, *sigB* for species-level discrimination, invasion-associated protein (*iap*) for additional species confirmation, and LIPI-1 alleles for detecting hallmark virulence determinants of *L. monocytogenes*. These markers enable differentiation of pathogenic and non-pathogenic *Listeria* species and together with chromogenic selective/differential media [16,17], support high-confidence identification of isolates, especially *L. monocytogenes*, recovered from complex wastewater matrices.

In this manuscript, the terms “pathogenic *L. monocytogenes*” and “non-pathogenic *L. innocua*” were used in the regulatory and food-safety sense, consistent with FDA and USDA-FSIS risk frameworks, in which *L. monocytogenes* is treated as a presumptive pathogen regardless of strain-specific phenotypic characterization.

## 2. Materials and Methods

### 2.1. Bacterial Strains, Growth Media and Conditions

Bacterial cultures used in this study were obtained from our laboratory culture collection or isolated from environmental sources as indicated in Table 1. *Listeria* strains were cultured in Tryptic Soy broth and plated on Tryptic Soy agar (TSB, TSA, Becton-Dickinson, Difco brand, Franklin Lakes, NJ, USA) at 30 °C. Tentative confirmation as *Listeria* spp. was obtained by enrichment in Demi-Fraser broth (BD-Difco) and streaking/plating on MOX agar base medium with supplements (BD-Difco). Additional methods of identification, including PCR bands on agarose gels, sequencing of PCR amplicons of various genes, and phenotypic reactions on *Listeria* media are explained below. Cultures were maintained for storage by centrifugation (6000× *g*, 5 °C) of 9 mL of fresh, overnight cultures and cell pellets were resuspended in 2–3 mL of fresh sterile TSB containing 10% glycerol. Cell suspensions were placed into glass vials and stored in an ultra-low freezer (−80 °C). Frozen stocks were revived by transferring 100 µL of the thawed cell suspension into 9 mL of TSB, incubating overnight at 30 °C, and sub-cultured again before use.

### 2.2. Isolation of Listeria spp. from Wastewater Using Anti-Listeria Immunomagnetic Beads

**Figure 1:**
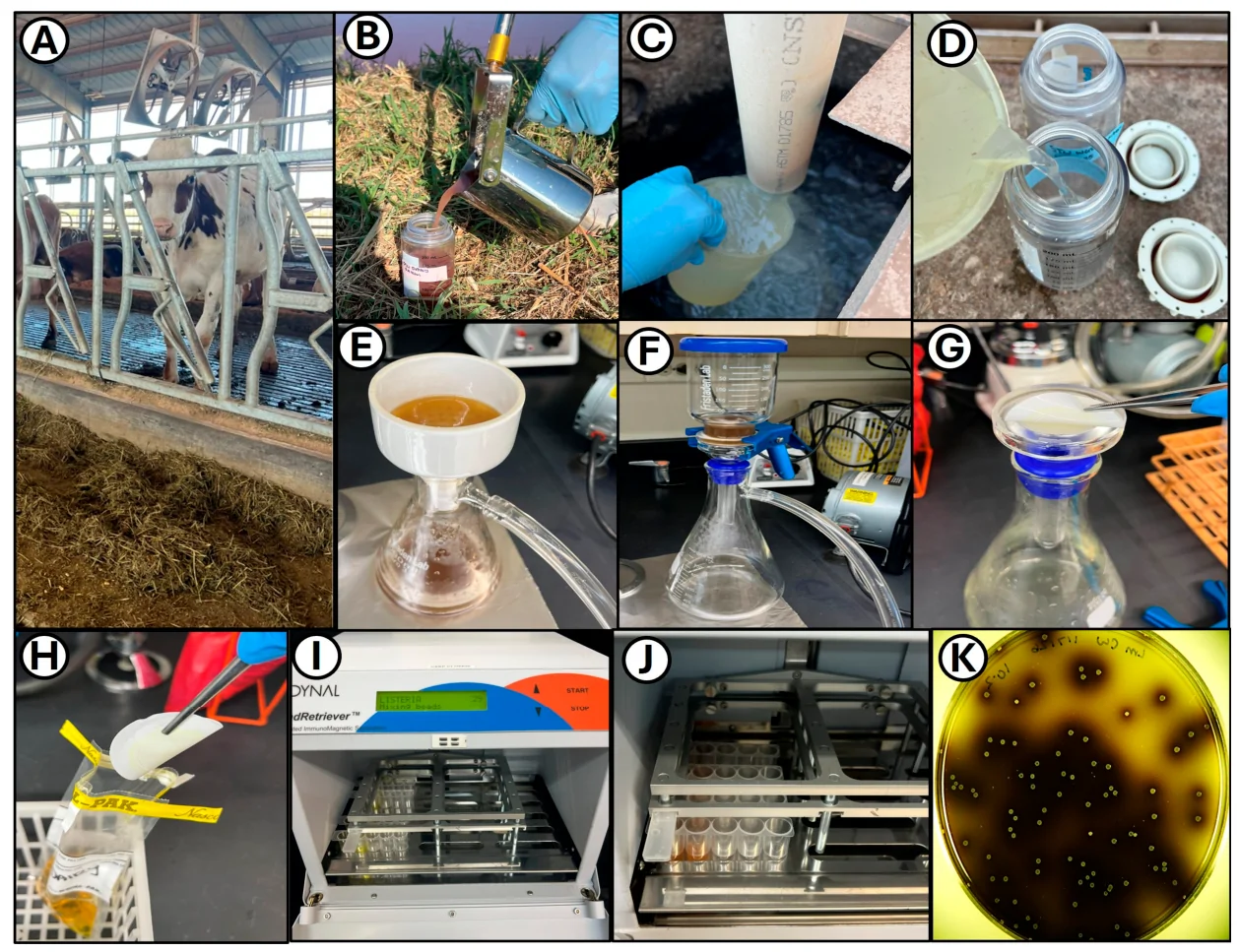
Sampling wastewater from OSU Dairy Center and municipal wastewater treatment center (Stillwater, OK). Panels: (**A**) OSU Dairy Center; (**B**) water collected from dairy center drainage into retention pond; (**C**) municipal wastewater supply (pre-treatment); (**D**) wastewater collection bottles; (**E**) Buchner funnel to filter particles; (**F**) 0.2-micron pore membrane filter to capture bacteria; (**G**,**H**) removal of membrane filter into enrichment broth bag; (**I**,**J**) BeadRetriever for programmed shaking, recovery, washing of immunomagnetic beads with *Listeria* captured from enriched membrane-filtered samples; (**K**) samples plated onto MOX agar plates with putative *Listeria* (black colonies).

Twenty-one (21) wastewater samples were collected from the city wastewater facility (13 samples) and the OSU Dairy Farm runoff lagoon (8 samples) (Figure 1A–C). The Stillwater wastewater treatment plant (Stillwater, OK, USA) had a convenient continuously running pipe from which raw wastewater could be sampled before treatment; the Oklahoma State University Dairy Farm runoff from dairy cattle facilities led to a retention pond and samples were retrieved using a telescoping bottle-holding device near the spillway; these samples contained a significant amount of suspended solids and fecal matter. Interest in wastewater runoff from the OSU Dairy Farm was heightened following the deaths of 6 unborn cattle from pregnant dairy cows due to *L. monocytogenes* in the preceding months that were attributed to contaminated silage. In either case, 200 mL water samples were recovered using large sterile plastic beakers to transfer to sterile 250 mL centrifuge tubes that were placed into a picnic cooler with ice packs, brought back to the lab and processed immediately, or refrigerated and processed the next day (Figure 1B,D).

Water samples that were extremely cloudy with suspended material (mostly dairy farm samples) were first filtered through sterile paper filters (20-micron pore size; StonyLab; Nesconset, NY, USA) using 80 mm Buchner funnels and a 1000 mL vacuum flask setup (Qwork, Hangzhou, Zhejiang Province, China) (Figure 1E). The filtrate was then filtered a second time through a sterile 47 mm 0.2-micron membrane filter that was removed and added to a sterile stomacher bag with 100 mL of Demi-Fraser broth enrichment media (Difco), incubated overnight at 30 °C, and sampled the next day (Figure 1F–H). After enrichment, a 1 mL sample was taken and processed with magnetic beads coated with anti-*Listeria* antibodies (Dynabeads anti*-Listeria*, Thermo Fisher Scientific, Waltham, MA, USA) using a ‘BeadRetriever’ (Dynal/Invitrogen, Carlsbad, CA, USA) for immunomagnetic separation (IMS) (Figure 1I,J). The BeadRetriever included program settings for recovery and washing *Listeria* cells adhered to paramagnetic beads covered with anti-*Listeria* antibodies. The process involves placing enrichment samples in initial cupules and adding Dynabeads with anti-*Listeria* antibodies, shaking to facilitate sample agitation, lowering the plastic sleeve-covered magnetic fingers into the sample, removing the magnetic beads from enrichment broth and dropping them into new cupules with buffer (phosphate-buffered saline, pH 7.4 with 0.05% Tween 20). Retraction of the magnetic fingers from the plastic sleeve causes beads to drop into the cupule. This was then followed by several cycles of washing with buffer (PBS + Tween) for final recovery of magnetic beads with adhered *Listeria*. The recovered *Listeria* samples were then streaked or plated onto MOX agar (Difco) and individual colonies were re-streaked and recovered for identity determination (Figure 1K). TS broth + 10% glycerol was added to residual captured-bead suspensions and stored at −80 °C for further sampling if needed. Additionally, enriched samples with recovered immunomagnetic beads (or freeze-stored samples) were plated or streaked on Brilliance Agar with appropriate supplements (Oxoid Brand, Thermo Fisher Scientific, Waltham, MA, USA) for putative determination as *L. monocytogenes*.

### 2.3. Phenotypic Reaction of Listeria spp. Isolated from Municipal and Dairy Center on Listeria Brilliance Agar

Oxoid *Listeria* Brilliance Agar (LBA, Thermo Fisher) is a selective and differential agar medium similar to Modified Oxford Media (MOX) having both selective and differential components. It has a base medium plus two supplements. One supplement contains four antibiotics (selective supplements) that provide selective recovery of *L. monocytogenes*/*Listeria* spp. and suppress other bacteria. The second supplement contains two differential components: an X-glucoside that is hydrolyzed by β-glucosidase to produce a chromogen making colonies appear blue-green, and a lecithin, which is acted upon by lecithinase (phospholipase) of *L. monocytogenes*/*L. ivanovii* to produce ‘white halos’ in the media surrounding the blue-green colonies. Blue-green colonies without white halos are neither *L. monocytogenes* nor *L. ivanovii*, but likely one of many other species of *Listeria*.

### 2.4. Aseptic Processing and Negative Controls

All filtration equipment (funnels, flasks, and membrane-filtration assemblies) were autoclaved prior to use. Samples were processed individually and completely through to enrichment, with no more than two wastewater samples handled per day. After each completed sample processing, all equipment was removed and the workspace, filtration station, and BeadRetriever were sanitized with a chlorine-based disinfectant before introducing a new set of sterile equipment. Filtered materials were transferred only into sterile bottles.

Sterile water was periodically run through the complete workflow as a negative control. These controls consistently produced no colonies on MOX agar, confirming that the sanitation procedures and workflow prevented cross-contamination.

### 2.5. Identity of Listeria monocytogenes and Listeria Species Isolated from Wastewater

Putative *Listeria* isolates obtained from typical reactions on MOX agar were examined by PCR sequencing of 16S [18], sigmaB (*sigB*) factor [19], and invasion-associated protein (*iap*) [13] to help establish identity based on molecular sequence determination. Additional analyses were obtained by agarose gel analysis of expected PCR reactions related to four of six genes involved with *Listeria monocytogenes* Pathogenic Island 1 gene cluster (LIPI-1: *hly*, *inlA*, *actA*, and *prfA* [15,20]). Phenotypic confirmation was further established by plating/streaking on *Listeria* Brilliance Agar [17] for reactions of β-glucosidase and phospholipase C.

#### 2.5.1. PCR and Sequence Analysis of 16S rRNA, sigB, and iap Genes of Isolates from Municipal and Dairy Wastewater

*Listeria* isolates from municipal and dairy center wastewater were cultured overnight in TSB (Difco) at 30 °C; the next day 1 mL of cell culture was micro-centrifuged and DNA was extracted from the cell pellet by a ‘bead beating’ technique [21,22,23] using 0.1 mm zirconia/silica disruption beads (RPI Research Products Intl., Mt. Prospect, IL, USA) and a bead tube holder/adapter fitted to a common laboratory vortexer (Takara, San Jose, CA, USA). After bead beating cell disruption, DNA samples were heated at 95 °C for 10 min in a thermal cycler. After cooling, DNA was then quantified using a Nanodrop ND-1000 spectrophotometer (ThermoFisher Scientific, Waltham, MA, USA) to determine template concentration. PCR was performed using primers determined within consensus sequence regions identified after multiple sequence alignments of the same genes from different isolates and primer selection using Vector NTI software (ver. 11.5; ThermoFisher) (Table 2). PCR reactions (25 μL) comprised: DNA template (5 μL, 1 ng/ μL), forward and reverse primers (10 μM, 1 μL each), GoTaq 2x MasterMix (12.5 μL; Promega, Madison, WI, USA). PCR conditions included initial denaturation (4 min at 95 °C), and then 40 cycles of: denaturation (30 s at 94 °C), annealing (30 s at 56 °C depending on T_m_ of primers), extension (1 min at 72 °C), melting curve analysis (50 °C to 90 °C, read every 0.2 °C, hold 6 min), and a final hold at 4 °C until retrieved. PCR was performed on an Opticon 2 thermal cycler (MJ Research, Waltham, MA, USA) running Opticon 3 software. Reactions with different primer annealing temperatures were run by virtue of an annealing temperature gradient across the plate.

After PCR, reactions were cleaned up using the IBI PCR Clean-Up kit (IBI Scientific, Dubuque, IA, USA). Briefly, a high salt ‘binding buffer’ is added to PCR reactions which are then loaded onto a DNA Filter Spin Column (glass fiber filter) to which DNA binds under high-salt conditions, centrifuged, and the eluate is discarded. The spin column is centrifuged again after each addition of 2 different wash buffers. Finally, centrifugation with a low salt Tris buffer releases and elutes DNA from the silica matrix. PCR reactions cleaned up in this manner were quantified by a Nanodrop spectrophotometer and 15 μL (15 ng/μL) was submitted to our OSU DNA Core Facility for DNA sequencing.

Various 16S rRNA ‘universal primers’ for bacteria have been well characterized [10,24]. Two sets of primers spanned nearly the entirety of the 16S rRNA region during culture-dependent microbiome analysis of bacteria isolated during biltong beef processing [18]. The numerical sequence of the various primers refers to their position in the generic *E. coli* K-12 16S rRNA reference sequence which may differ from the exact position in any other organism because of slight sequence differences.

Another region that is not as strictly conserved as 16S rRNA is polymerase sigma factor B (*sigB*; σ^b^) that is involved in RNA polymerase transcription of genes, but only for the specific set of promoters in its σ^b^ regulon. This region has also been used to identify bacterial species where 16S rRNA sequence differentiation is too constrained [19].

Lastly, when there is discordance between 16S rRNA and *sigB* species identity, a less-conserved allele is often used that offers additional mutational differences and species differentiation. Among *Listeria* species, the invasion-associated protein (*iap*, protein p60) is often used [13]. The name is a misnomer as it was originally thought it was involved with cell invasion but is now known to be involved with hydrolase activity during cell division in *Listeria*.

#### 2.5.2. Agarose Gel Analysis of *L. monocytogenes* Pathogenic Island (LIPI-1) Genes Involved in Pathogenesis

Stock cultures of pathogenic and non-pathogenic *Listeria* sp. were examined by PCR reactions with primers for four LIPI-1 genes (*hly*A, *inl*A, *prf*A, *act*A1; Table 2) and amplicons were run on 2% agarose gels (DNA grade agarose, Fisher Scientific) with 1x TAE buffer prepared from a 10x stock (Fisher Scientific). Gel wells were loaded with 10 μL of sample (3–6 μL nuclease-free water, 1 μL BlueJuice buffer, 1–3 μL PCR sample); a 100 bp DNA ladder (Promega, Madison, WI, USA) was also included for determining the correct size range of amplicons. Gels were electrophoresed at 80 V (constant voltage) for ~90 min with the blue dye indicating the extent of migration of small DNA. After electrophoresis, the agarose gel was stained with 100 mL of ethidium bromide solution (0.5 μg/mL; Fisher Scientific). EtBr-stained gels were destained by floating in a water tray on a slow-moving shaker for 25 min and visualized with a gel documentation system (UVP GelDoc-IT, UVP LLC, Upland, CA, USA).

### 2.6. Statistical Analysis

Non-parametric categorical tests were used because of the limited dataset (small sample size, presence/absence of *Listeria monocytogenes*, species identity issues, skewed distributions, and single- vs. dual-isolate recovery). All analyses were performed using SigmaPlot 13.0 (Systat Inc., Palo Alto, CA, USA). Prevalence estimation of *L. monocytogenes* at each site was calculated as the proportion of samples yielding at least one isolate of *L. monocytogenes*. Exact binomial 95% confidence intervals were generated for each site. Differences in detection frequency between dairy wastewater and municipal wastewater sites were evaluated using a 2 × 2 Fisher’s Exact Test, appropriate for small, expected counts. Species distribution (*L. monocytogenes*, *L. innocua*, *Listeria* sp.) between sites was evaluated using a 3 × 2 contingency table. SigmaPlot automatically selected Chi square or Fisher’s Exact Test (R × C) depending on expected cell counts. A *p* value < 0.05 was considered statistically significant.

## 3. Results and Discussion

### 3.1. Isolation of Listeria from Wastewater

#### Immunomagnetic Separation

Recovery of *Listeria* sp. was facilitated by enrichment of membrane filters in DemiFraser broth, recovery of *Listeria* from these enrichments using paramagnetic beads coated with anti-*Listeria* antibodies with the BeadRetriever, and plating on MOX agar (Figure 1E–K). Use of a Buchner funnel and large-porosity (20-microns) paper filters allowed removal of large particles from water with high levels of debris; the filtrate was then filtered on 0.2-micron filter membranes and bacteria trapped on their surface were enriched in Demi-Fraser selective broth. Commercial immunomagnetic beads for *Listeria* were only available with generic antibodies for *Listeria* sp., and not for *L. monocytogenes*, which would have been preferable. Enriched, bead-captured samples were plated directly on MOX or *Listeria* Brilliance agar immediately or stored frozen by addition of sterile TS broth/10% glycerol that is often used to freeze-store bacterial cultures, and plated later on Brilliance agar.

### 3.2. Phenotypic Reactions of Listeria spp. on Selective Media Isolated from Municipal and Dairy Center Environmental Sources of Wastewater

#### 3.2.1. Phenotypic Reactions on MOX Agar

*Listeria* sp. appear on MOX agar as dark colonies due to the hydrolysis of esculin in combination with ferric ammonium citrate to produce a black precipitate surrounding the colony. Some contaminants that grow up as MOX-reactive (darkened) colonies appear as normal convex colony morphology and can be distinguished from *Listeria* that appear as concave colonies. Using this IMS + MOX approach, typical *Listeria*-like colonies were isolated with every sample of municipal or dairy center wastewater examined; subsequent 16S rRNA sequencing confirmed these isolates as *Listeria* sp.

#### 3.2.2. Phenotypic Reactions on Listeria Brilliance Agar

**Figure 2:**
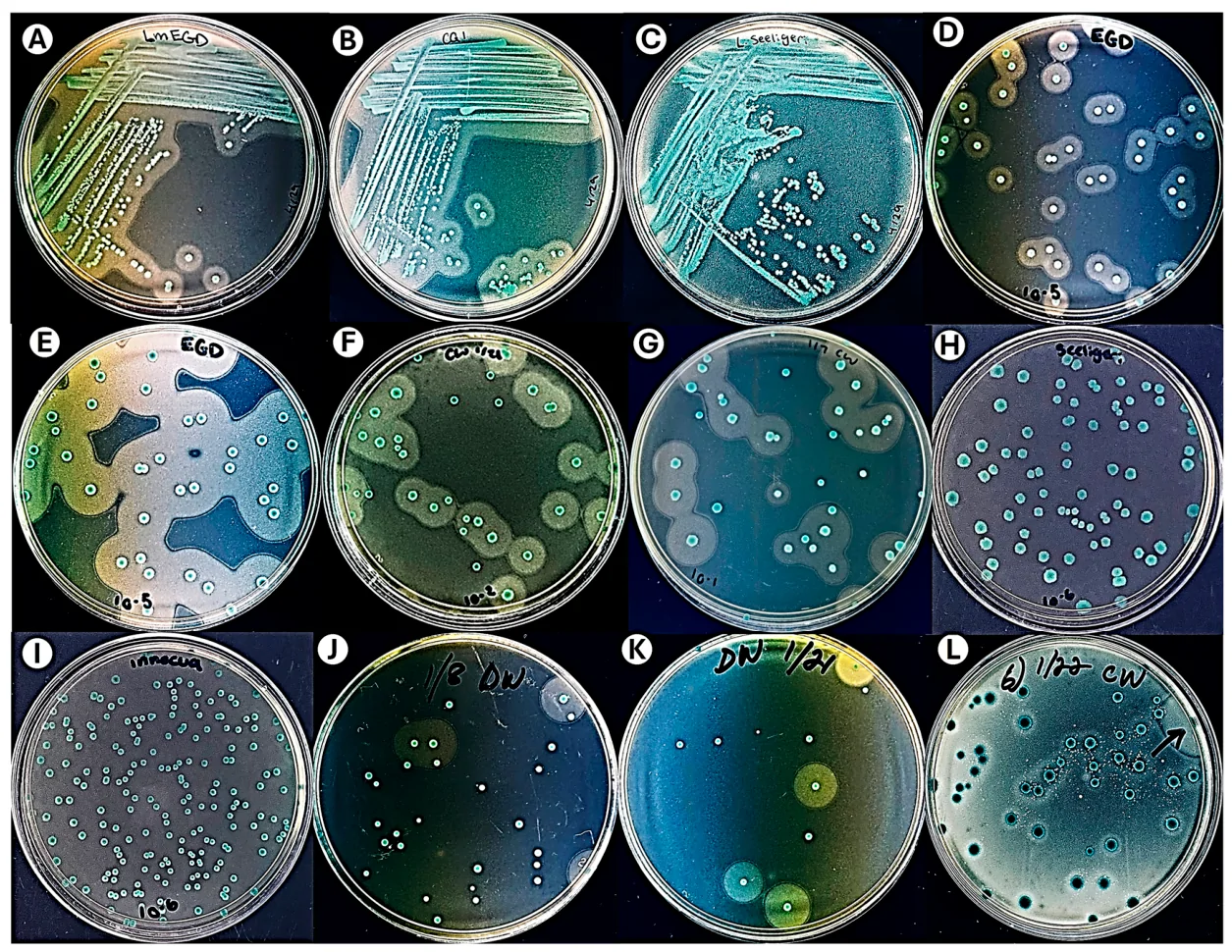
Reactions of stock *Listeria* strains and isolates on *Listeria* Brilliance agar. Streak plates, panels: (**A**) *L. monocytogenes* EGDe, (**B**) *L. monocytogenes* CQ1, and (**C**) *L. seeligeri* ATCC 35967. Surface spread plates, panels: (**D**,**E**) *L. monocytogenes* EGDe (48 and 72 h); (**F**,**G**) selective enrichment of IMS bead samples from municipal wastewater; (**H**,**I**) *L. seeligeri* ATCC 35967 and *L. innocua* ATCC 33090 (72 h); and selective enrichment of IMS samples from dairy (**J**,**K**) and city (**L**) wastewater.

Although MOX agar was useful for tentatively identifying *Listeria* sp., it does not differentiate between any of the species. One of the newer chromogenic agars (LL agar, Harlequin *Listeria* agar, ALOA agar, *Listeria* Brilliance agar) [17,25,26,27] was used that could distinguish putative *L. monocytogenes* from other species. Oxoid *Listeria* Brilliance agar (LBA) was selected because of its ability to distinguish *L. monocytogenes* and *L. ivanovii* by their ability to form “white halos” on the selective/differential agar medium. The halos form from the action of the phospholipase-mediated hydrolysis of phosphatidylcholine in the agar, producing white precipitated zones that increased in size with extended incubation surrounding blue-green colonies (Figure 2).

All remaining immunomagnetic bead samples previously screened on MOX agar and stored frozen were back-tested once Brilliance agar was received, resulting in the recovery of additional *L. monocytogenes* isolates that had not been detected on MOX agar with random colony testing. The added phenotypic discrimination of ‘white halo’ reaction with LBA was highly valuable in the tentative identification of *L. monocytogenes* that was subsequently confirmed by PCR and sequencing.

A key phenotypic marker supporting identification of *L. monocytogenes* in this process is the activity of phospholipase C, an enzyme used by this pathogen to dissolve phospholipid membranes, escape intracellular vesicles (i.e., endolysosomes, phagolysosomes) and mammalian host cells to facilitate intracellular survival [28,29]. *Listeria* Brilliance agar is one of several chromogenic media that have been developed that can differentiate *L. monocytogenes* (or *L. ivanovii*) from other *Listeria* sp. [25,26,30,31]. Furthermore, the chromogen X-glucoside is hydrolyzed by β-glucosidase to produce a greenish-blue colony color while the *L. monocytogenes*/*L. ivanovii* phospholipase C hydrolyzes the phosphatidylcholine (lecithin) to produce the characteristic white halo surrounding the colony (Figure 2). *Listeria* Brilliance agar was used to screen *Listeria* recovered by immunomagnetic separation (IMS) after filter-membrane enrichment (Figure 2).

### 3.3. Sequence Analysis of Control Strains and Putative Listeria Isolates from Wastewater

#### 3.3.1. Sequence Analysis of 16S rRNA Genes

The reason that the same 16S rRNA primers amplify many different bacterial taxa is that the 16S rRNA gene is functionally essential and highly conserved. Because ribosomal operation tolerates very little sequence variation, the gene exhibits limited divergence between bacterial genera, and even less among species within the same genus.

Following PCR amplification, sequencing, and BLAST (BLASTN 2.17.0+) analysis of the 16S rRNA gene, species-level identifications were obtained for several isolates reported as *Listeria cossartiae* (Table 3). Although such identifications would not be unusual for unknown and newly isolated strains, the same *L. cossartiae* identities were obtained using known ATCC reference cultures (Table 3). *L. cossartiae* is one of several new species recently described originating out of the Weidmann Lab (Cornell University, Ithaca, NY, USA) identified from agricultural water and environmental soil samples [32].

This outcome can be explained by the highly conserved nature of the 16S rRNA gene within *Listeria* spp. which prevents reliable species-level discrimination. *L. monocytogenes* has been shown to cluster with *L. cossartiae* and several other species in a category designated as the “most common ancestor” of *Listeria* sp. [33]. For this reason, a less-conserved genetic marker, the sigma factor B (or *sigB*) was examined as a possible species-identifying locus.

#### 3.3.2. Sequence Analysis of Listeria Sigma Factor B Genes

The Sigma B RNA polymerase factor (σ^B^) is a master regulator of the stress response in many bacteria, including *Listeria* [34]. It functions as an alternative subunit for RNA polymerase and is normally maintained in an inactive state by the “anti-sigma factor” RsbW. When bacteria encounter environmental stress (acid, salt, heat), a signaling cascade releases *sigB* enabling it to bind to the core RNA polymerase and direct transcription towards stress response promoters. Among *Listeria* sp., the *sigB* gene is moderately conserved and is typically found within the general stress response operon (*rsbV-rsbW-sigB-rsbX*) [35] and is often used for species-level identification in combination with other genetic loci [11,36].

In our study, identities provided by *sigB* sequencing differed markedly from those obtained using 16S rRNA. Most were identified as *L. innocua*, whereas seven isolates, as well as the known ATCC *L. monocytogenes* strains, were identified as *L. monocytogenes* (Table 3). Additional support for identification of *L. monocytogenes* was accommodated using the invasion-associated protein (*iap*) locus, which was expected to exhibit less conservation than either 16S or *sigB*, allowing the assessment of concordance across multiple loci.

#### 3.3.3. Sequence Analysis of *Listeria iap* Genes

The *Listeria* invasion-associated protein gene (*iap*) has also been used for species identification [13,37,38,39]. The *iap* gene encodes the p60 protein and is one of the most commonly employed single-gene markers for *Listeria* spp. because it contains conserved regions shared across the genus as well as variable regions allowing discrimination. Our approach was to use the conserved regions on either end of the *iap* gene as universal priming sites and use the variable internal region alongside 16S and *sigB* to confirm species identity.

Although the designation *iap* stood for “invasion-associated protein (p60)”, the gene actually encodes a cell wall hydrolase involved in cell division but the designation remained unchanged. Pilgrim et al. [40] had proposed renaming the gene cell wall hydrolase A (*cwh*A) to reflect its true function; however, the original designation has remained unchanged.

#### 3.3.4. PCR and Agarose Gel Electrophoresis of Amplicons Involved with *Listeria monocytogenes* Pathogenic Island 1 (LIPI-1) Genes

**Figure 3:**
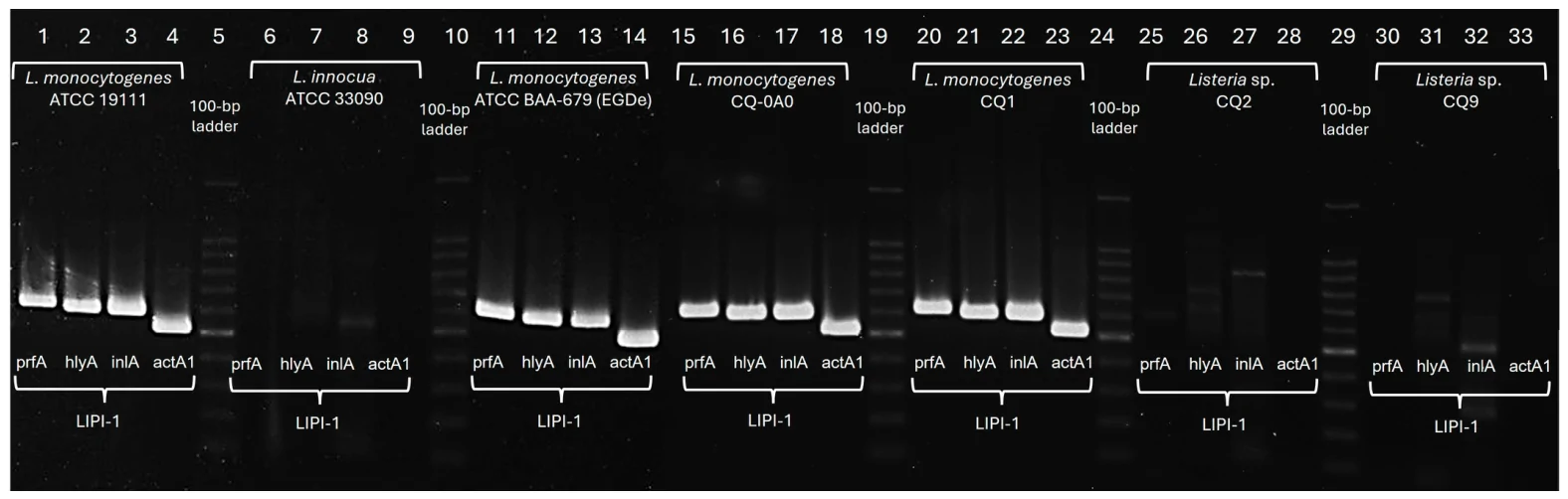
Ethidium bromide-stained agarose gels of PCR amplicons using primers for various LIPI-1 genes (positive regulatory factor A, *prf*A; hemolysin A, *hly*A; internalin A, *inl*A; and actin-based motility, *act*A, of various strains from our culture collection showing pathogenic strains (*L. monocytogenes* ATCC 19111 and EDGe) as well as non-pathogenic strains (*L. innocua* ATCC 33090, *L. seeligeri* ATCC 35967, and *L. welshimeri* ATCC 35897).

**Figure 4:**
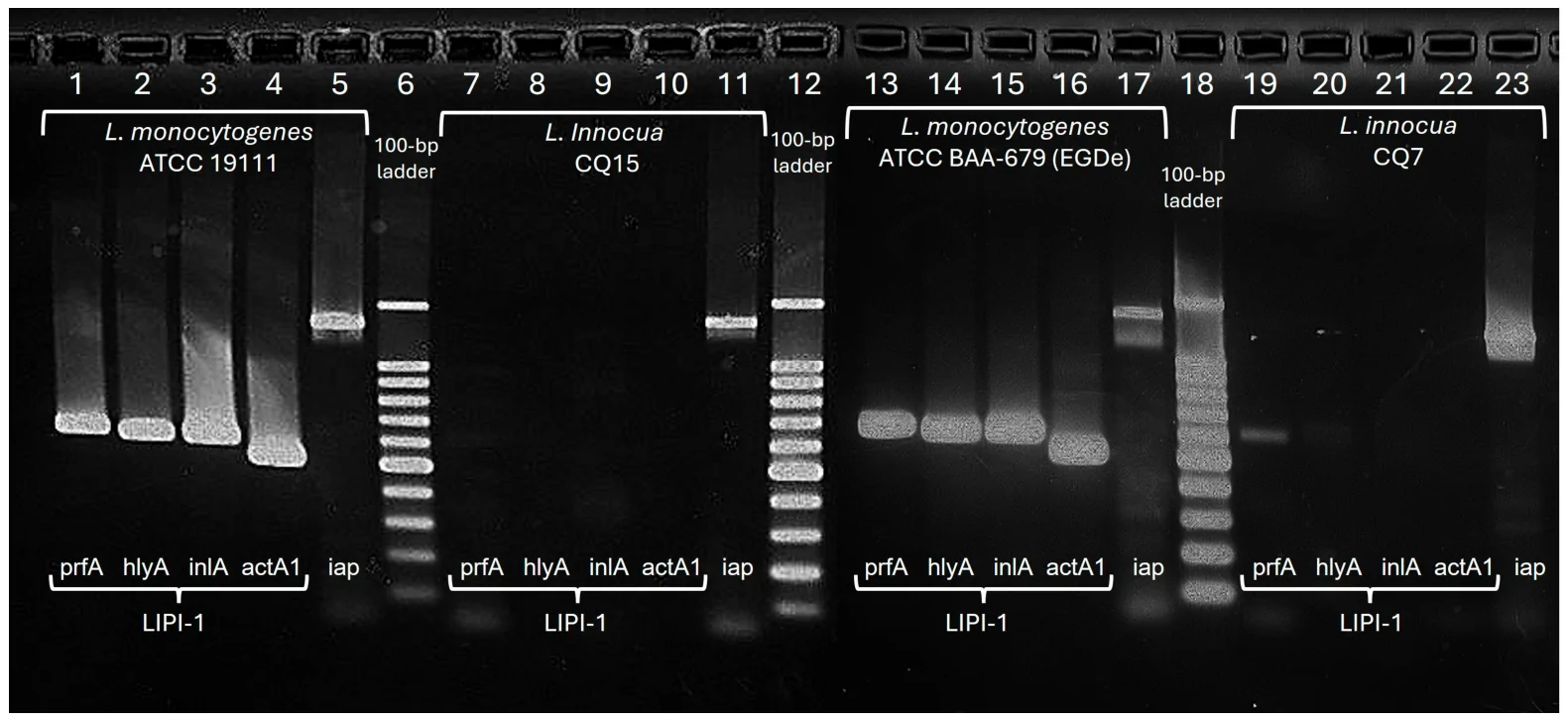
Agarose gel analysis of PCR products from additional wastewater isolates with primers for the four LIPI-1 genes used in this study including *iap*. The results indicate that CQ15 and CQ7 are non-pathogenic *Listeria innocua* as determined by *sigB* and *iap* sequence analysis.

**Figure 5:**
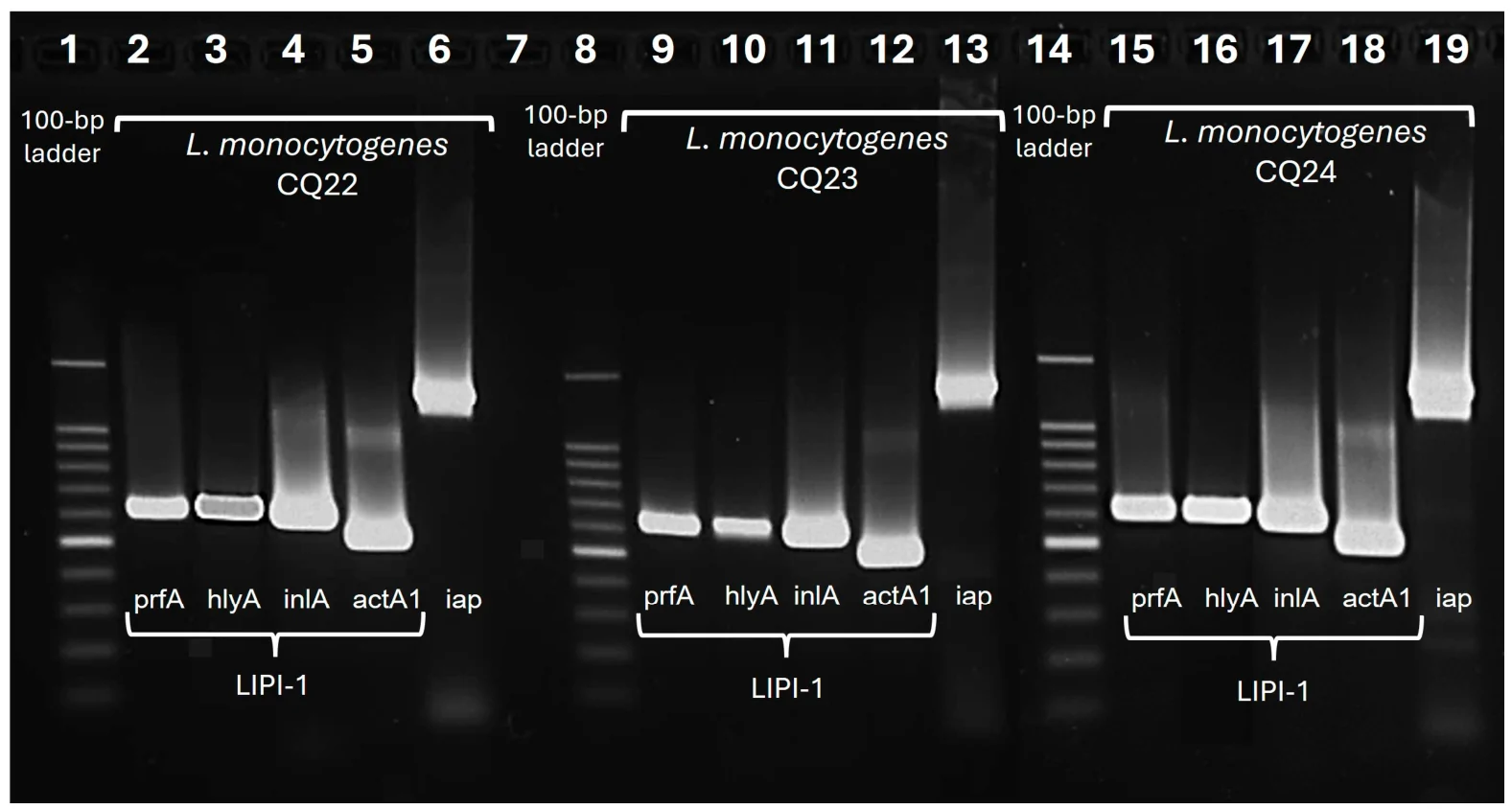
Agarose gels of PCR results using primers for *L. monocytogenes* Pathogenic Island 1 (LIPI-1) and *iap* against isolates obtained from municipal wastewater and OSU dairy wastewater (CQ22, CQ23, and CQ24).

Despite the strong agreement between *sigB* and *iap* to confirm species identity of *L. monocytogenes*, PCR amplicons representing *hly*A, *act*A1, *inlA*, and *prf*A were examined to verify the presence of these four LIPI-1 pathogenicity island genes. Amplicons of appropriate size were obtained for all four loci from each of the three ATCC *L. monocytogenes* reference strains as well as seven environmental isolates from dairy and municipal wastewater. In contrast, other *Listeria* spp. (*L. innocua*, *L. seeligeri*, and *L. welshimeri*) lacked these loci (Table 3, Figure 3, Figure 4 and Figure 5). It should be noted that the presence of virulence loci and even the identity as *L. monocytogenes* does not imply functional pathogenicity which has not been proven in this study.

PCR detection of the invasion-associated protein (*iap*, p60) was subsequently included on agarose gel analyses of amplicons. Isolates CQ0A0, CQ1, CQ19, CQ21, CQ22, CQ23, CQ24 were all determined to be *L. monocytogenes* based on the presence of all four LIPI-1 genes (and *iap*) while the other isolates predominantly showed only the *iap* amplicon. We also observed that the *iap* gene did not amplify in CQ14 and several other isolates that are confirmed as *Listeria* sp.; these samples were re-tested several times with the same results.

Initial PCR regimens used theoretical annealing temperatures based on the common guideline of using annealing temperatures 2–5 °C below the primer T_m_ [41,42,43]. This approach produced faint or smeared bands in some agarose gel lanes, which was interpreted as non-specific priming. Most non-pathogenic *Listeria* isolates produced cleaner PCR profiles after optimization of annealing temperatures to 2–4 °C above the primer T_m_, thereby reducing non-specific amplification artifacts [44]. This was subsequently verified by running an annealing temperature gradient across the thermal cycler’s 96-well plate with the same DNA template from an isolate giving spurious bands and smears [45]. Our ability to obtain suspect isolates that were later confirmed as *L. monocytogenes* was aided by the use of *Listeria* Brilliance agar, which produced the characteristic white halos described earlier.

### 3.4. Statistical Analysis of Listeria Isolates from Wastewater

A total of 27 *Listeria* isolates were recovered. Municipal wastewater yielded 16 isolates (5 *L. monocytogenes*, 6 *L. innocua*, 5 *Listeria* spp.), while dairy lagoon wastewater yielded 11 isolates (2 *L. monocytogenes*, 8 *L. innocua*, 1 *Listeria* sp.). Overall, *L. monocytogenes* represented 25.9% of all isolates while *L. innocua* represented 51.9% of all isolates.

Prevalence of *L. monocytogenes* was determined for the two testing sites. *L. monocytogenes* was detected in two of eight dairy samples (25%) and five of 13 municipal samples (38.5%). Exact binomial confidence intervals overlapped substantially (dairy: 7–52%; municipal: 17–64%), indicating similar prevalence across sites (Table 4). Fisher’s Exact Test showed no significant difference in *L. monocytogenes* detection between dairy and municipal wastewater (*p* > 0.05).

Both sites yielded mixed *Listeria* populations. Testing of dairy wastewater produced 2 isolates of *L. monocytogenes*, 8 of *L. innocua*, and 1 *Listeria* sp. Testing municipal wastewater produced 5 isolates of *L. monocytogenes*, 6 of *L. innocua*, and 5 *Listeria* sp. The 3 × 2 contingency test indicated no significant difference in species distribution between sites (*p* > 0.05) (Table 5).

The detection of multiple *Listeria* species across municipal and dairy wastewater samples reinforces the role of wastewater as an environmental reservoir for this genus. In this study, 27 isolates of *Listeria* were recovered from 21 samples inequitably taken from 2 sites, and several samples yielded more than one isolate. As a result, isolate counts do not directly reflect prevalence, which must be calculated based on the number of positive samples. When prevalence is evaluated correctly at the sample level, *L. monocytogenes* was detected in 33.3% of samples, with similar frequencies in municipal (38.5%) and dairy (25%) wastewater. This indicates that *L. monocytogenes* is consistently present but not dominant. *L. innocua* was detected in nearly half of all samples, suggesting broader distribution in these matrices. However, six isolates could not be resolved beyond *Listeria* sp., limiting the ability to draw strong conclusions about the relative abundance of specific species. These unresolved isolates highlight the challenges of species-level identification in environmental samples, where mixed populations, low biomass, or genomic similarity among *Listeria* spp. can impede definitive classification [46,47].

Historically, others have similarly noted a higher prevalence of *L. innocua* among a mixture of *Listeria* sp. Kaszoni-Rückerl et al. found a higher prevalence of *L. innocua* (*n* = 139) over *L. monocytogenes* (*n* = 81) in food (dairy) processing plants and showed that this was due to a growth advantage in selective and agar growth media [48]. In a comparative genomic study of 108 strains of *L. innocua* isolated from food and collected from four countries, other investigators found *L. innocua* to carry multiple stress systems grouped as Survival Island 2 and heavy-metal resistance genomic islands that give it a major ecological advantage in food and food environments [49]. These and other studies in comparative genomics highlight the fact that *L. innocua* lacks some of the major pathogenicity islands required for virulence while retaining stress-related determinants. Although there is no direct proof, this suggests that the reduced economic burden from the absence of virulence determinants provides a mechanistic basis for greater prevalence in food and food processing facilities as well as external natural environments.

## 4. Conclusions

This study shows that *Listeria* spp. are consistently present in municipal and dairy farm wastewater and that combining immunomagnetic separation, chromogenic media, and multilocus genetic markers enables reliable detection of *L. monocytogenes* in complex matrices. All wastewater samples were screened on *Listeria* Brilliance Agar, including magnetic bead–captured cells that were frozen and back-tested once the medium was received. Seven isolates were confirmed as *L. monocytogenes* through LIPI 1 amplification. Some limitations and gaps in the present study include isolation of species other than *L. monocytogenes* was not pursued for all samples (i.e., once Brilliance agar was implemented in the middle of the study) limiting the assessment of prevalence of *L. innocua* or other species. Additional gaps include the inability to adequately identify *Listeria* species for six of 27 isolates. Other ecological drivers for persistence were not identified, including the potential for biofilm formation on farm surfaces (dairy isolates), survival kinetics in silage vs. feed vs. water, and competition or synergy between *L. innocua* and *L. monocytogenes*. However, these were beyond the scope of the current project which was to isolate *L. monocytogenes* from wastewater as potential hosts for wastewater-derived bacteriophage.

Although six isolates could not be resolved to species level, the workflow effectively distinguished *L. monocytogenes* from other species. Broader ecological factors influencing persistence such as biofilm formation, survival in feed or silage, and interactions between *L. innocua* and *L. monocytogenes* remain to be investigated, as the primary aim was to obtain *L. monocytogenes* isolates for downstream bacteriophage enrichment.

These findings support wastewater monitoring as a complementary tool for tracking environmental dissemination of foodborne pathogens. Future work will assess whether internalin A-specific magnetic beads improve *L. monocytogenes* recovery and whether wastewater streams provide a reliable source of *L. monocytogenes*-specific bacteriophage. The isolates obtained here will be evaluated as candidate hosts for phage enrichment from the same wastewater sources.

## Figures and Tables

**Table 1:** *Listeria* strains from our culture collection and those isolated during this study.

Organism (Closest Species) ^1^	Strain Designation	Source
*Listeria monocytogenes*	EGDe; ATCC BAA-679	Muriana Culture Collection
*Listeria monocytogenes*	ScottA; ATCC 45954 45954	Muriana Culture Collection
*Listeria monocytogenes*	ATCC 19111	Muriana Culture Collection
*Listeria innocua*	ATCC 33090	Muriana Culture Collection
*Listeria seeligeri*	ATCC 35967	Muriana Culture Collection
*Listeria welshimeri*	ATCC 35897	Muriana Culture Collection
*Listeria monocytogenes*	CQ0A0	City Municipal Wastewater
*Listeria* spp.	CQ0A1	City Municipal Wastewater
*Listeria* spp.	CQ0A2	City Municipal Wastewater
*Listeria monocytogenes*	CQ1	City Municipal Wastewater
*Listeria* spp.	CQ2	City Municipal Wastewater
*Listeria innocua*	CQ3	OSU Dairy Wastewater
*Listeria innocua*	CQ4	OSU Dairy Wastewater
*Listeria innocua*	CQ5	OSU Dairy Wastewater
*Listeria innocua*	CQ6	OSU Dairy Wastewater
*Listeria innocua*	CQ7	City Municipal Wastewater
*Listeria innocua*	CQ8	City Municipal Wastewater
*Listeria* spp.	CQ9	City Municipal Wastewater
*Listeria innocua*	CQ10	City Municipal Wastewater
*Listeria innocua*	CQ11	OSU Dairy Wastewater
*Listeria innocua*	CQ12	OSU Dairy Wastewater
*Listeria innocua*	CQ13	City Municipal Wastewater
*Listeria* spp.	CQ14	City Municipal Wastewater
*Listeria innocua*	CQ15	OSU Dairy Wastewater
*Listeria innocua*	CQ16	OSU Dairy Wastewater
*Listeria innocua*	CQ17	City Municipal Wastewater
*Listeria innocua*	CQ18	City Municipal Wastewater
*Listeria monocytogenes*	CQ19	City Municipal Wastewater
*Listeria* spp.	CQ20	OSU Dairy Wastewater
*Listeria monocytogenes*	CQ21	City Municipal Wastewater
*Listeria monocytogenes*	CQ22	OSU Dairy Wastewater
*Listeria monocytogenes*	CQ23	OSU Dairy Wastewater
*Listeria monocytogenes*	CQ24	City Municipal Wastewater

^1^ Note: Species identity was based on a combination of 16S, *sigB*, *iap*, and/or *L. monocytogenes* Pathogenic Island PCR (LIPI-1) genes and response to *Listeria* Brilliance Agar phenotype.

**Table 2:** PCR primers used in this study.

Primer Pairs	Target Gene	Primer Sequence (5′→3′)	Product Size (bp)	(NCBI) Primer T_m_ (°C)	(IDT) Primer T_m_ (°C)
Primer 1	Hemolysin (*hly*A)				
	Forward	TGAACCTACAAGACCTTCCA	560	55.7	53.0
	Reverse	CAATTTCGTTACCTTCAGGA		53.2	50.1
Primer 2	Internalin A (*inl*A)				
	Forward	GCTTCAGGCGGATAGATTAG	575	55.5	52.6
	Reverse	AACTCGCCAATGTGCC		54.7	53.3
Primer 3	Positive regulatory factor (*prf*A)				
	Forward	ATTTTTAACCAATGGGATCC	590	51.2	48.2
	Reverse	CATTCATCTAATTTAGGGGC		51.3	48.3
Primer 4	Actin mobility (*act*A1)				
	Forward	AATACGAACAAAGCAGACCTAATAG	500	57.2	52.9
	Reverse	GGTCAATTAACCCTGCACTTTTA		57.3	53.5
Primer 6	Universal 16 S rRNA—front half				
	Forward (7F)	RAGAGTTTGATCHTGGCTCAG	~920–930	54	53.5
	Reverse (928R)	CCCCGTCAATTCHTTTGA		47	50.8
Primer 7	Universal 16 S rRNA—back half				
	Forward (759F)	CAGGATTAGATACCCTGGTAGTCC	~782	59.2	55.8
	Reverse (1541R)	AAGGAGGTGATCCARCCGC		59.7	58.8
Primer 8	Sigma Factor B (*sigB*)				
	Forward	AAAGCAGGTGGAGGAGAATG	~749	57.5	54.8
	Reverse	TTGACGTTGGATTCTAGACACAT		57.9	54.0
Primer 9	Invasion-Associated Protein (*iap*)				
	Forward	GAATATGAAAAAAGCAACTATCGC	~1286	51.6	51.1
	Reverse	CTAAATCACCAGGTTTTGCTTG		51.1	52.4

**Table 3:** Genetic sequence and phenotypic analysis of Listeria isolates recovered from municipal and OSU dairy center wastewater.

Organism	Strain Designation	Source ^1^	Date Obtained	16S rRNA Seq. ID (Closest Species)	16S Seq. (bp)	16S nBlast %Query; %ID	*SigB* Seq. ID (Closest Species)	*SigB* Seq. (bp)	*SigB* nBlast %Query; %ID	*Iap* Seq. ID (Closest Species)	*Iap* Seq. (bp)	*Iap* nBlast % Query; % ID	Lm Pathogenic Island PCR Results (Y/N) ^2^				Reaction on *Listeria* Brilliance Agar ^3^
													*prfA*	*hlyA*	*actA1*	*inlA*	
*L. monocytogenes*	EGDe (ATCC-BAA-679)	Cult. Coll.	N/A	*L. cossartiae*	1519	100; 99.8	*L. monocytogenes*	729	100;100	*L. monocytogenes*	1098	100; 99.9	Y	Y	Y	Y	Halo
*L. monocytogenes*	Scott-A (ATCC 45954)	Cult. Coll.	N/A	*L. cossartiae*	1521	100; 99.7	*L. monocytogenes*	730	100; 99.9	*L. monocytogenes*	1109	100; 100	Y	Y	Y	Y	Halo
*L. monocytogenes*	ATCC 19111	Cult. Coll.	N/A	*L. cossartiae*	1518	100; 99.7	*L. monocytogenes*	730	100;100	*L. monocytogenes*	1100	100; 100	Y	Y	Y	Y	Halo
*L. innocua*	ATCC 33090	Cult. Coll.	N/A	*L. welshimeri*	1522	100; 99.6	*L. innocua*	730	100; 100	*L. innocua*	1157	100; 100	N	N	N	N	No Halo
*L. seeligeri*	ATCC 35967	Cult. Coll.	N/A	*L. welshimeri*	1522	100; 99.5	*L. innocua*	730	100; 99.9	*L. innocua*	1178	100; 100	N	N	N	N	No Halo
*L. welshimeri*	ATCC 35897	Cult. Coll.	N/A	*L. welshimeri*	1521	100; 99.9	*L. welshimeri*	730	100; 99.9	*L. innocua*	1171	100; 99.5	N	N	N	N	No Halo
*L. monocytogenes*	CQ-0A0	City W.W.	8 December 2025	*L. cossartiae*	1518	100; 99.4	*L. monocytogenes*	727	100; 100	*L. monocytogenes*	1090	94; 96.2	Y	Y	Y	Y	Halo
*Listeria* sp.	CQ-0A1	City W.W.	11 December 2025	*L. cossartiae*	1519	100; 99.6	*L. innocua*	737	100; 99.3	*L. welshimeri*	954	100; 100	N	N	N	N	No Halo
*Listeria* sp.	CQ-0A2	City W.W.	16 December 2025	*L. welshimeri*	1520	100; 99.6	*L. monocytogenes*	680	100; 99.9	*L. innocua*	822	100; 99.4	N	N	N	N	No Halo
*L. monocytogenes*	CQ1	City W.W.	7 January 2026	*L. cossartiae*	1522	100; 99.7	*L. monocytogenes*	729	100; 100	*L. monocytogenes*	1166	100; 99.8	Y	Y	Y	Y	Halo
*Listeria* sp.	CQ2	City W.W.	7 January 2026	*L. cossartiae*	907	100; 99.6	*L. innocua*	680	100; 99.7	Not determined			N	N	N	N	No Halo
*L. innocua*	CQ3	OSU Dairy	7 January 2026	*L. cossartiae*	902	100; 99.3	*L. innocua*	730	100; 99.9	*L. innocua*	893	100; 99.8	N	N	N	N	No Halo
*L. innocua*	CQ4	OSU Dairy	7 January 2026	*L. cossartiae*	1521	100; 99.6	*L. innocua*	729	100; 100	*L. innocua*	1137	100; 100	N	N	N	N	No Halo
*L. innocua*	CQ5	OSU Dairy	8 January 2026	*L. cossartiae*	919	100; 99.1	*L. innocua*	724	100; 100	*L. innocua*	1091	100; 100	N	N	N	N	No Halo
*L. innocua*	CQ6	OSU Dairy	8 January 2026	*L. cossartiae*	918	100; 99.2	*L. innocua*	729	100; 100	*L. innocua*	991	100; 99.4	N	N	N	N	No Halo
*L. innocua*	CQ7	City W.W.	8 January 2026	*L. cossartiae*	919	100; 99.4	*L. innocua*	727	100; 99.6	*L. innocua*	932	100; 99.9	N	N	N	N	No Halo
*L. innocua*	CQ8	City W.W.	8 January 2026	*L. cossartiae*	880	100; 99.3	*L. innocua*	725	100; 99.7	*L. innocua*	1050	100; 100	N	N	N	N	No Halo
*Listeria* sp.	CQ9	City W.W.	14 January 2026	*L. cossartiae*	861	100; 99.2	*L. innocua*	730	100; 99.9	Not determined			N	N	N	N	No Halo
*L. innocua*	CQ10	City W.W.	14 January 2026	*L. cossartiae*	901	100; 99.7	*L. innocua*	729	100; 99.9	*L. innocua*	1121	100; 99.0	N	N	N	N	No Halo
*L. innocua*	CQ11	OSU Dairy	14 January 2026	*L. welshimeri*	906	100; 99.5	*L. innocua*	731	100; 99.2	*L. innocua*	851	100; 99.7	N	N	N	N	No Halo
*L. innocua*	CQ12	OSU Dairy	14 January 2026	*L. cossartiae*	873	100; 99.2	*L. innocua*	729	100; 100	*L. innocua*	1143	100; 99.7	N	N	N	N	No Halo
*L. innocua*	CQ13	City W.W.	21 January 2026	*L. cossartiae*	874	100; 99.4	*L. innocua*	730	100; 99.3	*L. innocua*	1115	100; 99.8	N	N	N	N	No Halo
*Listeria* sp.	CQ14	City W.W.	21 January 2026	*L. cossartiae*	904	100; 99.8	*L. innocua*	731	100; 99.9	Not determined			N	N	N	N	No Halo
*L. innocua*	CQ15	OSU Dairy	21 January 2026	*L. cossartiae*	903	100; 99.2	*L. innocua*	730	100; 100	*L. innocua*	1084	100; 99.6	N	N	N	N	No Halo
*L. innocua*	CQ16	OSU Dairy	21 January 2026	*L. cossartiae*	903	100; 99.7	*L. innocua*	703	100; 99.9	*L. innocua*	1027	100; 99.2	N	N	N	N	No Halo
*L. innocua*	CQ17	City W.W.	22 January 2026	*L. innocua*	865	100; 99.5	*L. innocua*	730	100; 100	*L. innocua*	780	100; 100	N	N	N	N	No Halo
*L. innocua*	CQ18	City W.W.	22 January 2026	*L. cossartiae*	904	100; 99.3	*L. innocua*	729	100; 100	*L. innocua*	803	100; 99.5	N	N	N	N	No Halo
*L. monocytogenes*	CQ19	City W.W.	7 January 2026	*L. cossartiae*	906	100; 99.6	*L. monocytogenes*	728	100; 100	*L. monocytogenes*	1207	100; 99.8	Y	Y	Y	Y	Halo
*Listeria* sp.	CQ20	OSU Dairy	7 January 2026	*Ent. saccharolyticus*	914	100; 99.8	*L. innocua*	751	100; 99.6	No reaction			N	N	N	N	No Halo
*L. monocytogenes*	CQ21	City W.W.	21 January 2026	*L. cossartiae*	904	100; 99.6	*L. monocytogenes*	730	100; 100	*L. monocytogenes*	1063	100; 100	Y	Y	Y	Y	Halo
*L. monocytogenes*	CQ22	OSU Dairy	8 January 2026	*L. cossartiae*	910	100; 99.5	*L. monocytogenes*	730	100; 100	*L. monocytogenes*	1126	100; 100	Y	Y	Y	Y	Halo
*L. monocytogenes*	CQ23	OSU Dairy	21 January 2026	*L. cossartiae*	910	100; 99.5	*L. monocytogenes*	729	100; 100	*L. monocytogenes*	1164	100; 100	Y	Y	Y	Y	Halo
*L. monocytogenes*	CQ24	City W.W.	22 January 2026	*L. cossartiae*	911	100; 99.3	*L. monocytogenes*	729	100; 100	*L. monocytogenes*	1118	100; 100	Y	Y	Y	Y	Halo

^1^ Sources: Cult. Coll.: Culture Collection; City W.W.: City Wastewater; OSU Dairy: OSU Dairy Wastewater. ^2^ *Listeria monocytogenes* Pathogenic Island 1 (LIPI-1) results of representative strains are demonstrated in the accompanying agarose gel figures. Absence of the 4 distinct bands is strong evidence that the species is other than *L. monocytogenes*’. ^3^ Reactions on *Listeria* Brilliance Agar: *Listeria* spp. produce blue-green colonies due to β-glucosidase activity on X-glucoside; *L. monocytogenes* (& *L. ivanovii*) produce a white ‘halo’ due to lecithinase activity (halo = presumptive *L. monocytogenes*).

**Table 4:** Prevalence of *L. monocytogenes* in wastewater samples.

Site	Total Samples	Lmono- Positive	Prevalence (%)	95% Confidence Interval *
Dairy	8	2	25.0	7–52
Municipal	13	5	38.5	17–64

* Due to the small and unequitable sample size from each site, exact binomial confidence intervals overlapped substantially (dairy: 7–52%; municipal: 17–64%), indicating similar prevalences across sites.

**Table 5:** Distribution of *Listeria* sp. among wastewater testing sites.

Species	Dairy (*n* = 11 Isolates)	Municipal (*n* = 16 Isolates)
*L. monocytogenes*	2	5
*L. innocua*	8	6
*Listeria* sp.	1	5

## Data Availability

The original contributions presented in this study are included in the article. Further inquiries can be directed to the corresponding author.
